# Oxidative Stress Biomarkers Predict Myocardial Dysfunction in a Chemotherapy-Induced Rat Model

**DOI:** 10.3390/diagnostics15060705

**Published:** 2025-03-12

**Authors:** So Ree Kim, Dong-Hyuk Cho, Jong-Ho Kim, Seong-Mi Park, Mi-Na Kim

**Affiliations:** Division of Cardiology, Korea University Anam Hospital, Seoul 02841, Republic of Korea

**Keywords:** biomarkers, cardiomyopathy, chemotherapy, oxidative stress

## Abstract

**Objectives:** Chemotherapy improves survival in breast cancer patients but increases the risk of myocardial dysfunction and heart failure. Since early prediction of cardiomyopathy remains difficult, biomarkers are needed for detecting myocardial damage before heart failure develops. This study examines the association between oxidative stress biomarkers and myocardial dysfunction in a chemotherapy-induced rat model. **Methods:** Forty-two rats were randomized into four groups: control (*n* = 7), doxorubicin only (*n* = 7), doxorubicin plus trastuzumab (*n* = 7), and doxorubicin plus trastuzumab with cardioprotective intervention (*n* = 21). Doxorubicin and trastuzumab were administered sequentially over 28 days. Echocardiography with speckle-tracking was utilized to measure longitudinal strain (LS, −%). Reduced LS was defined by a LS with a median value less than 23% on day 28. Blood samples were collected for biomarker analysis, focusing on superoxide dismutase (SOD) and glutathione (GSH). Myocardium fibrosis was assessed using Masson’s trichrome staining. **Results:** Thirty-four rats survived and underwent LS analysis. All rats treated with doxorubicin and trastuzumab exhibited reduced LS, while those receiving cardioprotective intervention maintained preserved LS on day 28. The reduced LS group had significantly lower SOD and higher GSH levels compared to the preserved LS group. SOD and GSH correlated strongly with LS (SOD, r = 0.590, *p* = 0.001; GSH, r = −0.590, *p* = 0.003), and LS correlated with fibrosis area (r = −0.660, *p* < 0.001). SOD and GSH effectively predicted reduced LS. **Conclusions:** In a rat model of chemotherapy-induced cardiomyopathy, oxidative stress biomarkers correlated with myocardial dysfunction, as indicated by LS. These findings highlight the potential of biomarker monitoring to improve early detection and prevention strategies for chemotherapy-induced cardiomyopathy.

## 1. Introduction

Breast cancer survivors have a high incidence of cardiovascular diseases (CVDs), with many ultimately succumbing to CVDs rather than cancer [1]. Women with pre-existing risk factors such as hypertension and diabetes are particularly vulnerable to developing overt CVDs when exposed to cardiotoxic treatments. Doxorubicin is known to induce various cardiac complications, ranging from asymptomatic left ventricular (LV) dysfunction to overt heart failure [2]. Additionally, while trastuzumab enhances overall survival in patients with human epidermal growth factor receptor 2-positive breast cancer, its concurrent use with doxorubicin intensifies cardiotoxic effects [3].

There are currently no reliable methods for the early detection of cardiomyopathy [4]. Myocardial strain showed promising data [5,6], while recent studies have failed to demonstrate the benefits of strain-guided cardioprotection in mitigating LV ejection fraction (EF) reduction and the development of cancer therapeutics-related cardiac dysfunction (CTRCD) in high-risk patients undergoing chemotherapy [7]. In addition, resource limitations restrict the frequent use of echocardiography. N-terminal pro-brain natriuretic peptide (NT-proBNP) can be normal in patients having silent myocardial damage, because it increases with stretching of the myocardial wall. Therefore, other biomarkers are needed for the early detection of myocardial damage before overt heart failure development and to guide cardioprotective treatment.

Oxidative stress indicators can be reduced by administration of cardiotoxic agents [8] and several animal studies demonstrated their associations with myocardial dysfunction [9,10]. However, little is known for the correlation between oxidative stress indicators and myocardial strain in chemotherapy-induced cardiomyopathy models. This study explores the association between oxidative stress biomarkers, including superoxide dismutase (SOD, enzymatic) and glutathione (GSH, non-enzymatic), and myocardial dysfunction in a chemotherapy-induced rat model.

## 2. Materials and Methods

Forty-two rats were randomly assigned to four groups. Group 1 (*n* = 7) served as the control group receiving no drugs. Group 2 (*n* = 7) received doxorubicin alone (cumulative dose of 20 mg/kg). Group 3 (*n* = 7) received both doxorubicin and trastuzumab (cumulative dose of 30 mg/kg), representing the chemotherapy-induced cardiomyopathy model. Group 4 (*n* = 21) received doxorubicin and trastuzumab along with cardioprotective interventions, consisting of three subgroups (*n* = 7 each): rosuvastatin alone, candesartan alone, or their combination. Conventional and two-dimensional (2D) speckle-tracking echocardiography were performed at baseline and on day 28. After completing the 28-day drug administration period, the rats were anesthetized, and blood samples were collected from the inferior vena cava via open thoracotomy. Following blood collection, rats were sacrificed, and their hearts were harvested for histopathological analysis to assess the degree of myocardial fibrosis and apoptosis. The detailed protocols were described in the previous report [11].

### 2.1. Animals and Study Protocols

Five-week-old female Sprague Dawley rats underwent a two-week acclimatization period before this study commenced. A long-term in vivo rat model of left ventricular (LV) dysfunction was established through sequential intraperitoneal injections of doxorubicin and trastuzumab. Doxorubicin was administered intraperitoneally every other day for two weeks, followed by trastuzumab injections on the same schedule for an additional two weeks. Rosuvastatin (10 mg/kg) and/or candesartan (0.5 mg/kg) were administered daily via gastric lavage for four weeks, starting from day one. The study protocols were approved by the Korea University Institutional Ethics Committee for Animal Research (KOREA-2016-0244) and adhered to the U.S. National Institutes of Health guidelines on animal protection in scientific research.

### 2.2. Echocardiography

Rats were lightly sedated and imaging was performed using the Vivid-E95 (GE Vingmed, Horten, Norway) equipped with an 18 MHz linear probe designed for animal research. The imaging depth was set to 2 cm, and both parasternal long-axis and short-axis views were obtained. All echocardiographic assessments and data collection were performed by a single cardiologist (D-H.C.), who has conducted over 300 rat echocardiographic examinations.

### 2.3. LV Longitudinal Strain by 2D Speckle-Tracking

Two-dimensional cine loops were acquired at a frame rate of over 80 frames per second, covering at least five consecutive cardiac cycles in the parasternal long-axis view. Longitudinal strain (LS) was evaluated using 2D speckle-tracking software (ECHOPAC PC, ver. 201.67.0, GE Vingmed, Horten, Norway), with manual delineation of the endocardial and epicardial borders. Measurements were averaged over five heartbeats. Rats with an LS < 23%, which is the median value of the LS on day 28, were considered the reduced LS group, while the others were classified as the preserved LS group. All echocardiographic assessments were conducted by echocardiographers who were blinded to the study groups. All strain data were described using the absolute LS value.

### 2.4. Biochemical Evaluation

On day 28, blood samples were collected. The collected samples were centrifuged at 2200 rpm for 10 min at 4 °C. Serum levels of troponin I, NT-proBNP, and C-reactive protein (CRP) were measured using enzyme-linked immunosorbent assay (ELISA) kits (Cloud-Clone, Katy, TX, USA). To evaluate oxidative stress markers, the activity of SOD, as well as serum levels of GSH, nitric oxide, and reactive oxygen species (ROS), were assessed according to the manufacturer’s protocols.

### 2.5. Histopathological Analysis

Immediately after blood was sampled, the rats were euthanized, and their hearts were collected. Masson’s trichrome stain was performed using a trichrome stain kit (HT15, Sigma Aldrich, Burlington, MA, USA) to assess the extent of myocardial fibrosis, following the manufacturer’s guidelines. Apoptosis was quantified using the terminal deoxynucleotidyl transferase-mediated deoxyuridine triphosphate nick-end labeling (TUNEL) assay with the APO-BrdU TUNEL assay kit (A23210, Invitrogen, Waltham, MA, USA). Digital images were acquired using an epi-fluorescence system at 400× magnification and analyzed by a blinded observer (J-H.K.).

### 2.6. Statistical Analysis

Continuous variables were presented as the mean ± standard deviation, while categorical variables were expressed as percentages. Differences in parameters between groups were compared by the chi-square test or Student’s *t*-test. The assumption of normality was tested by the Shapiro–Wilk test and a Q–Q plot. Correlation analysis was performed to explore the relationship between myocardial dysfunction and oxidative stress markers or histopathological measurements using Pearson’s correlation. Receiver operating characteristic curves for predicting reduced LS using oxidative stress markers were evaluated. A two-tailed *p* value of <0.05 was considered statistically significant. SPSS version 24.0 software (IBM, Armonk, NY, USA) and R version 4·3·1 (R Foundation for Statistical Computing, Vienna, Austria) were used for statistical analyses.

## 3. Results

**Figure 1:**
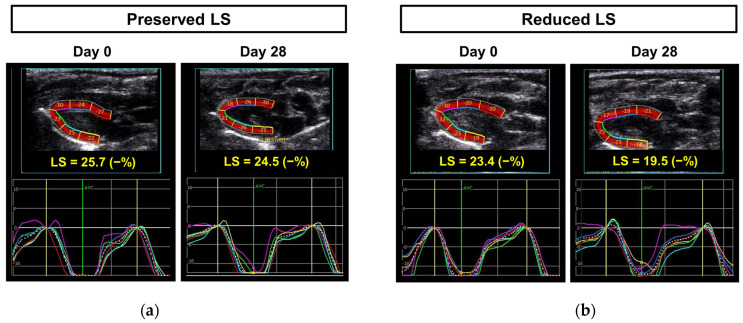
Representative cases of left ventricular LS based on preserved or reduced LS on day 28: (**a**) A case of preserved LS in a rat treated with doxorubicin, trastuzumab, rosuvastatin, and candesartan; (**b**) A case of reduced LS in a rat treated with doxorubicin and trastuzumab. LS, longitudinal strain.

**Figure 2:**
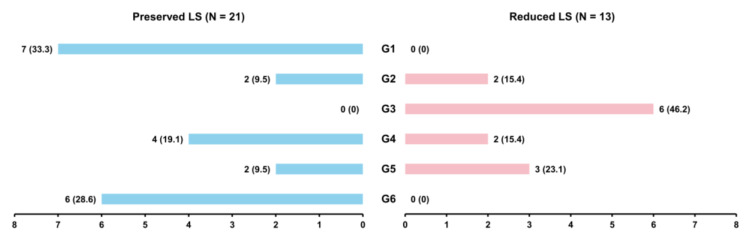
Distribution of groups based on preserved or reduced LS on day 28. G1, control; G2, doxorubicin only; G3, doxorubicin + trastuzumab; G4, doxorubicin + trastuzumab + rosuvastatin; G5, doxorubicin + trastuzumab + candesartan; G6, doxorubicin + trastuzumab + rosuvastatin + candesartan; LS, longitudinal strain.

Among the 34 rats that survived and underwent LS analysis on day 28, 13 exhibited a reduced LS of less than 23% (Figure 1). LS was preserved in all control rats and those treated with both rosuvastatin and candesartan (Figure 2). In contrast, all rats receiving doxorubicin and trastuzumab demonstrated a decline in LS on day 28. There were no differences in baseline weight or heart rate between the preserved and reduced LS groups (Table 1).

Echocardiographic data are summarized in Table 1. Baseline measurements, including LV chamber size and FS, were similar between the two groups, as was the initial LS. However, on day 28 the reduced LS group exhibited a significantly larger chamber size and decreased FS compared to the preserved LS group. The LS values for each group were as follows: 20.7% ± 1.2% for the reduced LS group and 24.7% ± 1.1% for the preserved LS group (*p* < 0.001). Additionally, the change in LS (ΔLS) was significantly lower in the reduced LS group compared to the preserved LS group (−11.5% ± 7.7% vs. 4.2% ± 11.0%, *p* < 0.001).

**Figure 3:**
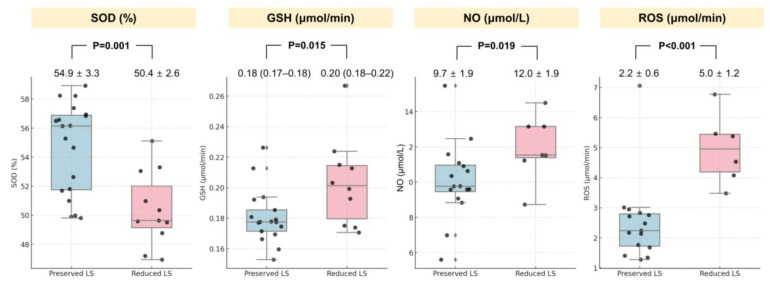
Oxidative stress biomarkers based on preserved or reduced LS on day 28. GSH, glutathione; NO, nitric oxide; ROS, reactive oxygen species; SOD, superoxide dismutase.

Biomarkers and histopathological findings are presented in Table 2 and Figure 3. On day 28, the reduced LS group had significantly higher levels of troponin I, NT-proBNP, and CRP compared to the preserved LS group. SOD levels were markedly lower in the reduced LS group (50.4 ± 2.6 vs. 54.9 ± 3.3, *p* < 0.001), whereas GSH levels were higher (0.20 [0.18–0.22] vs. 0.18 [0.17–0.18], *p* = 0.015). Furthermore, the reduced LS group exhibited greater myocardial fibrosis and apoptosis, as indicated by increased TUNEL staining.

**Figure 4:**
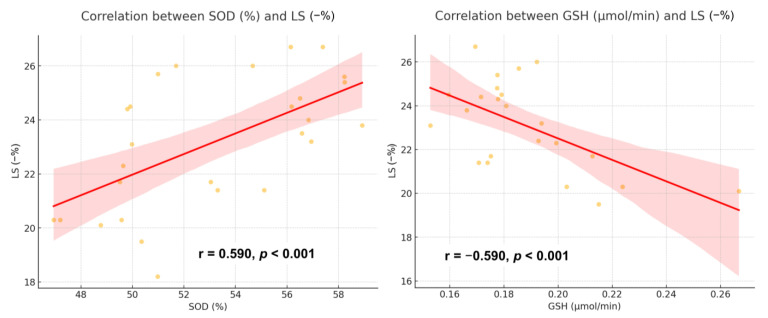
Correlation between oxidative stress biomarkers and LS. Red line: a linear regression line. Red shaded region: 95% confidence interval. LS, longitudinal strain.

**Figure 5:**
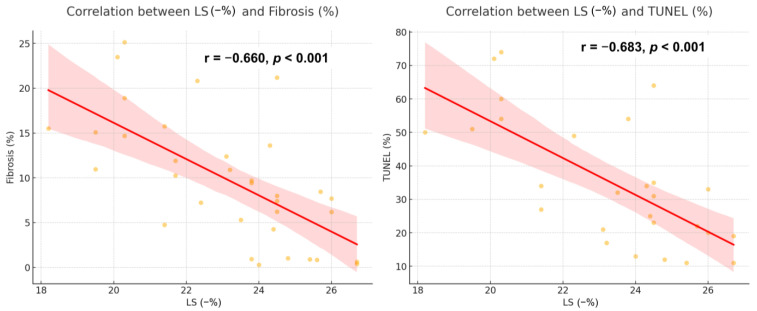
Correlation between LS and histopathological data. Red line: a linear regression line. Red shaded region: 95% confidence interval. LS, longitudinal strain.

**Figure 6:**
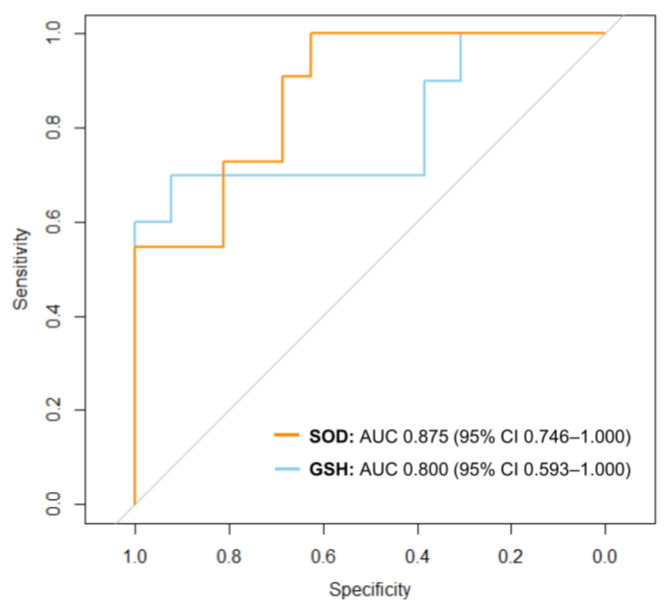
ROC curves to predict reduced LS by SOD and GSH. Gray line: a diagonal reference line. AUC, area under the curve; CI, confidence interval; GSH, glutathione; LS, longitudinal strain; SOD, superoxide dismutase; ROC, receiver operating characteristic.

Oxidative stress biomarkers exhibited a strong association with myocardial dysfunction. SOD correlated positively with LS (r = 0.590, *p* = 0.001), whereas GSH showed a negative correlation with LS (r = −0.590, *p* = 0.003) (Figure 4). Additionally, LS was significantly linked to myocardial fibrosis (r = −0.660, *p* < 0.001) and the extent of apoptosis (r = −0.683, *p* < 0.001) (Figure 5). Predictive analysis indicated that SOD could identify reduced LS with an area under the curve (AUC) of 0.875 (95% CI 0.746–1.000), while GSH predicted reduced LS with an AUC of 0.800 (95% CI 0.593–1.000) (Figure 6).

## 4. Discussion

This study investigates the relationship between oxidative stress biomarkers and myocardial dysfunction in a chemotherapy-induced rat model. All rats treated with doxorubicin and trastuzumab exhibited reduced LS, while those treated with cardioprotective intervention maintained preserved LS on day 28. The reduced LS group had significantly lower SOD levels and higher GSH levels compared to the preserved LS group. Oxidative stress biomarkers, including SOD and GSH, demonstrated a strong correlation with markers of myocardial dysfunction. Additionally, LS correlated with the fibrosis area of myocardium. Furthermore, SOD and GSH effectively predicted the probability of reduced LS.

Myocardial strain serves as a valuable tool for detecting early myocardial damage following chemotherapy, offering insights beyond LV ejection fraction [5,6]. Unlike traditional methods, speckle-tracking strain is independent of imaging angles and aligns well with myocardial fiber orientation [12]. Although recent studies have failed to demonstrate the benefits of LS-guided cardioprotection in mitigating LVEF reduction and the development of CTRCD in high-risk patients undergoing chemotherapy [7], LS remains essential for defining CTRCD, assessing baseline and follow-up cardiac function, and detecting subclinical myocardial dysfunction [13]. Speckle-tracking-based myocardial strain analysis is applicable to small-animal studies [14]. Notably, LS (expressed as a negative value) demonstrated a strong inverse relationship with myocardial fibrosis. This association underscores the importance of LS as a sensitive and innovative marker in this cardiomyopathy model.

SODs catalyze the conversion of superoxide into oxygen and hydrogen peroxide. They limit the potential toxicity of ROS and reactive nitrogen species and regulate the signaling pathways by controlling the levels of these molecules [15]. In a previous animal study using isolated cardiac myocytes, doxorubicin stimulated superoxide anion formation, and a decreased level of intracellular SOD activity made the myocytes more vulnerable to doxorubicin toxicity [16]. Furthermore, SOD inhibits doxorubicin-induced iron release from ferritin, leading to increased intracellular iron levels and enhanced toxicity [17]. Trastuzumab also induces early changes in the levels of oxidative stress biomarkers, decreasing SOD activity [8].

GSH is a vital intracellular antioxidant that helps maintain the cellular redox balance and protects cells from damage induced by lipid peroxides, ROS, and nitrogen species. Recent studies have highlighted its significant role in key signal transduction pathways, where it regulates processes such as cell differentiation, proliferation, apoptosis, ferroptosis, and immune function. Notably, GSH has both protective and detrimental effects: it detoxifies carcinogens in healthy cells but contributes to tumor progression and increased chemotherapy resistance in cancer cells [18]. Doxorubicin has been shown to deplete intracellular GSH levels [16]. However, previous studies indicated that GSH mitigates doxorubicin-induced myocardial toxicity [19,20]. GSH exerts its protective function by reducing ROS levels and modulating the pERK signaling pathway, as demonstrated in studies using human progenitor cells [21]. In the present study, unexpectedly, the reduced LS group exhibited higher glutathione levels than the preserved LS group. This may be explained by the body’s upregulation of glutathione synthesis as a protective response to early myocardial stress or damage. However, in cardiomyopathy an increased amount of glutathione may not be efficiently utilized due to mitochondrial dysfunction or impaired glutathione reductase activity.

Limited data are available on the correlation between oxidative stress biomarkers and myocardial dysfunction. In an animal study investigating the effect of SOD deficiency on exposure to pressure overload, extracellular SOD-deficient mice exhibited greater ventricular fibrosis, dilatation, and a more pronounced reduction in LV systolic function compared to wild-type mice [10]. Similarly, a study examining the relationship between chronic GSH depletion and pressure overload-induced cardiac failure in mice found that GSH-deficient mice experienced exacerbated LV remodeling and dysfunction under pressure overload, similar to the effects observed in SOD deficiency [9]. In a clinical study evaluating the acute cardiac toxicity of adjuvant trastuzumab, decreased activity of SOD was correlated with LVEF [8]. In addition, in a clinical study involving patients undergoing cardiac surgery, both cardiac and systemic GSH deficiency were associated with the severity of functional status, and atrial GSH levels correlated with LVEF, particularly in patients with coronary artery disease [22]. In contrast to these previous studies, the present study explores the relationship between oxidative stress biomarkers and LS, an early indicator of subclinical myocardial dysfunction, in the context of chemotherapy-induced cardiomyopathy.

This study has several limitations. First, myocardial dysfunction was assessed using an LS value of 23%. However, the optimal LS cut-off for defining myocardial dysfunction in an animal chemotherapy-induced cardiomyopathy model remains unclear. This study determined this threshold based on the median LS value on day 28 among surviving rats. Since three rats died before reaching day 28, the cut-off for reduced LS may have been overestimated. Nevertheless, speckle-tracking echocardiography effectively measured myocardial deformation, demonstrating both the cardiotoxic effects of doxorubicin and trastuzumab and the beneficial effects of cardioprotective intervention. Second, while numerous cardiomyopathy biomarkers are applicable to in vitro studies, this study only evaluated serum biomarkers. Measuring biomarkers at the molecular level could provide deeper insights into the mechanisms of myocardial dysfunction under chemotherapy. However, serum biomarkers such as troponin I, NT-proBNP, CRP, SOD, and GSH supported the observed cardiotoxicity of doxorubicin and trastuzumab. Third, the doses of these chemotherapeutic agents were considerably higher than those commonly used in clinical practice. To more accurately reflect human chemotherapy regimens, further long-term animal studies incorporating clinically relevant drug dosages and cardioprotective treatments are needed. Additionally, clinical study is needed before applying these findings to human patients.

## 5. Conclusions

The combination of doxorubicin and trastuzumab led to a significant reduction in LS, whereas the addition of cardioprotective intervention helped restore LS. Oxidative stress biomarkers, including SOD and GSH, exhibited a strong correlation with myocardial dysfunction, as indicated by LS. Moreover, SOD and GSH effectively predicted the risk of reduced LS. These findings suggest that monitoring these biomarkers could enhance early detection and prevention strategies for chemotherapy-induced cardiomyopathy.

## Figures and Tables

**Table 1:** Baseline characteristics and echocardiographic data at baseline and on day 28.

	Preserved LS (*n* = 21)	Reduced LS (*n* = 13)	*p*
Baseline			
Weight, g	248.5 ± 11.8	251.1 ± 6.3	0.398
Heart rate, bpm	208 (181–247)	183 (165–206)	0.111
LVDd, mm	62.5 ± 7.4	65.9 ± 4.3	0.148
LVSd, mm	23.5 ± 3.7	25.8 ± 3.1	0.077
FS, %	62.1 ± 6.0	60.9 ± 4.1	0.523
LS, −%	23.8 ± 1.9	23.7 ± 1.5	0.883
Day 28			
Weight, g	267.1 ± 22.0	246.2 ± 18.1	0.007
Heart rate, bpm	198 (183–222)	189 (180–197)	0.141
LVDd, mm	67.4 ± 4.2	71.0 ± 4.7	0.028
LVSd, mm	29.2 ± 4.1	36.6 ± 5.0	<0.001
FS, % *	56.8 ± 4.4	48.5 ± 5.6	<0.001
LS, −%	24.7 ± 1.1	20.7 ± 1.2	<0.001
%Change in LS ^†^	4.2 ± 11.0	−11.5 ± 7.7	<0.001

FS, fractional shortening; LS, longitudinal strain; LVDd, left ventricular end-diastolic dimension; LVSd, left ventricular end-systolic dimension. * FS = (LVDd—LVSd)/LVDd. ^†^ %Change in LS = ([LS at week 4—LS at baseline]/LS at baseline) ∗ 100%.

**Table 2:** Biomarkers and histopathological data on day 28.

	Preserved LS (*n* = 21)	Reduced LS (*n* = 13)	*p*
Biomarkers			
Troponin I, pg/mL	14.1 (13.4–16.1)	15.6 (15.1–17.8)	0.041
NT-proBNP, pg/mL	59.0 ± 6.4	78.2 ± 12.7	0.002
CRP, pg/mL	182.5 ± 70.6	277.3 ± 44.0	0.001
Histopathological data			
Fibrosis, %	6.5 ± 5.4	15.0 ± 6.0	<0.001
TUNEL, %	26.5 ± 14.4	52.3 ± 15.5	<0.001

CRP, C-reactive protein; NT-proBNP, N-terminal pro-brain natriuretic peptide; TUNEL, terminal deoxynucleotidyl transferase-mediated deoxyuridine triphosphate nick-end labeling.

## Data Availability

The data presented in this study are available on request from the corresponding author due to institutional restrictions related to animal research.

## Abbreviations

The following abbreviations are used in this manuscript:

CRP	C-reactive protein
CTRCD	Cancer therapeutics-related cardiac dysfunction
CVD	Cardiovascular disease
EF	Ejection fraction
GSH	Glutathione
LS	Longitudinal strain
LV	Left ventricle/left ventricular
NT-proBNP	N-terminal pro-brain natriuretic peptide
ROS	Reactive oxygen species
SOD	Superoxide dismutase
TUNEL	Terminal deoxynucleotidyl transferase-mediated deoxyuridine triphosphate nick-end labeling
2D	Two-dimensional

## References

[1] Yeo Y.H., San B.J., Tan J.Y., Tan M.C., Donisan T., Lee J.Z., Franey L.M., Hayek S.S. (2024). Cardiovascular mortality trends and disparities in U.S. breast cancer patients, 1999–2020: A population-based retrospective study. Cardiooncology.

[2] Camilli M., Cipolla C.M., Dent S., Minotti G., Cardinale D.M. (2024). Anthracycline Cardiotoxicity in Adult Cancer Patients: JACC: CardioOncology State-of-the-Art Review. JACC Cardio Oncol..

[3] Eaton H., Timm K.N. (2023). Mechanisms of trastuzumab induced cardiotoxicity—Is exercise a potential treatment?. Cardiooncology.

[4] Abdel-Qadir H., Nolan M.T., Thavendiranathan P. (2016). Routine Prophylactic Cardioprotective Therapy Should Be Given to All Recipients at Risk of Cardiotoxicity From Cancer Chemotherapy. Can. J. Cardiol..

[5] Sartorio A., Cristin L., Pont C.D., Farzaneh-Far A., Romano S. (2025). Global longitudinal strain as an early marker of cardiac damage after cardiotoxic medications, a state of the art review. Prog. Cardiovasc. Dis..

[6] Azzam M., Wasef M., Khalaf H., Al-Habbaa A. (2023). 3D-based strain analysis and cardiotoxicity detection in cancer patients received chemotherapy. BMC Cancer.

[7] Negishi T., Thavendiranathan P., Penicka M., Lemieux J., Murbraech K., Miyazaki S., Shirazi M., Santoro C., Cho G.Y., Popescu B.A. (2023). Cardioprotection Using Strain-Guided Management of Potentially Cardiotoxic Cancer Therapy: 3-Year Results of the SUCCOUR Trial. JACC Cardiovasc. Imaging.

[8] Dirican A., Levent F., Alacacioglu A., Kucukzeybek Y., Varol U., Kocabas U., Senoz O., Emre S.V., Demir L., Coban E. (2014). Acute cardiotoxic effects of adjuvant trastuzumab treatment and its relation to oxidative stress. Angiology.

[9] Watanabe Y., Watanabe K., Kobayashi T., Saito Y., Fujioka D., Nakamura T., Obata J.E., Kawabata K., Mishina H., Kugiyama K. (2013). Chronic depletion of glutathione exacerbates ventricular remodelling and dysfunction in the pressure-overloaded heart. Cardiovasc. Res..

[10] Lu Z., Xu X., Hu X., Zhu G., Zhang P., van Deel E.D., French J.P., Fassett J.T., Oury T.D., Bache R.J. (2008). Extracellular superoxide dismutase deficiency exacerbates pressure overload-induced left ventricular hypertrophy and dysfunction. Hypertension.

[11] Cho D.H., Lim I.R., Kim J.H., Kim M.N., Kim Y.H., Park K.H., Park S.M., Shim W.J. (2020). Protective Effects of Statin and Angiotensin Receptor Blocker in a Rat Model of Doxorubicin- and Trastuzumab-Induced Cardiomyopathy. J. Am. Soc. Echocardiogr..

[12] Gorcsan J., Tanaka H. (2011). Echocardiographic assessment of myocardial strain. J. Am. Coll. Cardiol..

[13] Lyon A.R., Lopez-Fernandez T., Couch L.S., Asteggiano R., Aznar M.C., Bergler-Klein J., Boriani G., Cardinale D., Cordoba R., Cosyns B. (2022). 2022 ESC Guidelines on cardio-oncology developed in collaboration with the European Hematology Association (EHA), the European Society for Therapeutic Radiology and Oncology (ESTRO) and the International Cardio-Oncology Society (IC-OS). Eur. Heart J..

[14] Koshizuka R., Ishizu T., Kameda Y., Kawamura R., Seo Y., Aonuma K. (2013). Longitudinal strain impairment as a marker of the progression of heart failure with preserved ejection fraction in a rat model. J. Am. Soc. Echocardiogr..

[15] Wang Y., Branicky R., Noe A., Hekimi S. (2018). Superoxide dismutases: Dual roles in controlling ROS damage and regulating ROS signaling. J. Cell Biol..

[16] Shi S., Chen Y., Luo Z., Nie G., Dai Y. (2023). Role of oxidative stress and inflammation-related signaling pathways in doxorubicin-induced cardiomyopathy. Cell Commun. Signal..

[17] Ru Q., Li Y., Chen L., Wu Y., Min J., Wang F. (2024). Iron homeostasis and ferroptosis in human diseases: Mechanisms and therapeutic prospects. Signal Transduct. Target Ther..

[18] Kennedy L., Sandhu J.K., Harper M.E., Cuperlovic-Culf M. (2020). Role of Glutathione in Cancer: From Mechanisms to Therapies. Biomolecules.

[19] Villani F., Galimberti M., Monti E., Piccinini F., Lanza E., Rozza A., Favalli L., Poggi P., Zunino F. (1990). Effect of glutathione and N-acetylcysteine on in vitro and in vivo cardiac toxicity of doxorubicin. Free Radic. Res. Commun..

[20] Fu X., Eggert M., Yoo S., Patel N., Zhong J., Steinke I., Govindarajulu M., Turumtay E.A., Mouli S., Panizzi P. (2020). The Cardioprotective Mechanism of Phenylaminoethyl Selenides (PAESe) Against Doxorubicin-Induced Cardiotoxicity Involves Frataxin. Front. Pharmacol..

[21] Lee E.J., Jang W.B., Choi J., Lim H.J., Park S., Rethineswaran V.K., Ha J.S., Yun J., Hong Y.J., Choi Y.J. (2023). The Protective Role of Glutathione against Doxorubicin-Induced Cardiotoxicity in Human Cardiac Progenitor Cells. Int. J. Mol. Sci..

[22] Damy T., Kirsch M., Khouzami L., Caramelle P., Le Corvoisier P., Roudot-Thoraval F., Dubois-Rande J.L., Hittinger L., Pavoine C., Pecker F. (2009). Glutathione deficiency in cardiac patients is related to the functional status and structural cardiac abnormalities. PLoS ONE.

